# Hemiarthroplasty compared to total hip arthroplasty for the treatment of femoral neck fractures: a systematic review and meta-analysis

**DOI:** 10.1186/s13018-020-02186-4

**Published:** 2021-03-03

**Authors:** Xinbo Li, Jianning Luo

**Affiliations:** 1 Trauma Orthopedics Ward 1, Ningbo No. 6 Hospital, No. 1059, Zhongshan East Road, Dongliu Street, Yinzhou District, Ningbo City, 315040 Zhejiang Province China; 2 Trauma Orthopedics Ward 2, Ningbo No. 6 Hospital, No. 1059, Zhongshan East Road, Dongliu Street, Yinzhou District, Ningbo City, 315040 Zhejiang Province China

**Keywords:** Hemiarthroplasty, Total hip arthroplasty, Femoral neck fractures, Meta-analysis

## Abstract

**Background:**

Hip replacement is divided into total hip arthroplasty (THA) and hemiarthroplasty (HA); it is still controversial whether to choose THA or HA for femoral neck fractures (FNF). The goal of this study was to review relevant studies in order to determine the HA compared to THA for the treatment of FNF.

**Patients and methods:**

Using appropriate keywords, we identified relevant studies using PubMed, Cochrane, and Embase. Key pertinent sources in the literature were also reviewed, and all articles published through August 2019 were considered for inclusion. For each study, we assessed odds ratios (ORs), mean difference (MD), and 95% confidence interval (95% CI) to assess and synthesize outcomes.

**Results:**

We included 19 studies with a total of 413,140 patients in the HA group and 44973 in the THA group. The blood loss, surgery time, and dislocation were all significantly decreased in the HA group than the THA group. The length of hospital, pneumonia, and renal failure were significant increased in the HA group than THA group. There has no significant difference of complication, mortality, reoperation, infection, pulmonary embolism, and myocardial infarct between the two groups.

**Conclusion:**

HA has favor in decrease blood loss and surgery time. THA has favor in decrease the length of hospital, the incidence of pneumonia and renal failure. For the selection of surgical methods for femoral neck fracture in the elderly, we should consider several aspects, such as the age of the patient, whether there is osteoporosis, the type of femoral neck fracture, the preoperative reduction situation, and the needs of the patient and his family for the postoperative situation.

**Supplementary Information:**

The online version contains supplementary material available at 10.1186/s13018-020-02186-4.

## Introduction

FNF accounts for about 3.6% of adult fractures, which is one of the more common fractures in the body. FNF is more common in elderly patients, generally refers to the fracture in the part of the femoral head down to the base of the femoral neck. Garden classification is often adopted for FNF in the elderly, type I and type II, because no displacement or displacement of fracture end to a lesser degree, damage degree of fracture is lesser, belongs to the stable fractures. Type III and type IV due to shifting more of fracture end, fracture damage is bigger, belongs to the unstable fractures. The clinical features and fracture healing of the four types has presented in Table 1 [1–3].

**Table 1:** The Garden classification of FNF

Garden classification of FNF	Clinical features of the fracture	Fracture healing
Type I	The fracture line does not run through the entire femoral neck, part of the bone is connected, and the broken end of the fracture is not displaced.	There is still a certain blood supply near the broken end, and the fracture is easy to heal.
Type II	Complete fracture with fracture line running through the neck of femur without displacement of the broken end.	Even if the fracture line is in good alignment under the femoral head, the possibility of fracture healing is higher, but the probability of long-term femoral head necrosis is increased. However, the fracture of the middle or basal part of the femoral neck is easy to heal, the femoral head blood supply is good, and the avascular necrosis of the femoral head is low.
Type III	Partial displacement of fracture, mostly distal upward displacement, or distal lower Angle embedded into the proximal folded end of the section.	It is an unstable fracture, postoperative fracture healing rate is not high.
Type IV	Fracture completely shift, femoral neck appear obvious outward turning up, the hip joint capsule and synovial suffered severe damage, femoral head poor blood supply	Fracture is not easy to heal, easy femoral head necrosis.

FNF in older patients are not stable fractures (Garden III, IV), accompanied with the complications: the serious situation of displacement fracture, the longer time of conservative treatment in bed, pulmonary embolism, falling pneumonia, lower limb thrombosis, bone nonunion, urinary system infection, and so on. At the same time, the conservative treatment of fracture carries the bad counterpoint to the line, high rate of fracture malunion, physical disability, ischemic necrosis of femoral head; so still need hip replacement later. Hip replacement is divided into THA and HA, THA requires replacement of femoral head and acetabulum. Compared with THA, HA only replaces the femoral head, which requires less technical requirements for the surgeon. The advantages of HA including less surgical trauma, less blood loss, and low economic cost; the disadvantages are high incidence of postoperative pain and further wear of untreated acetabular cartilage. Therefore, it is still controversial whether to choose THA or HA for specific patients [4, 5].

The aim of this study was to perform a meta-analysis of all available literature to obtain updated evidence to eradicate the HA versus THA for the treatment of FNF and to provide a basis for the selection of clinical treatment.

## Methods

### Search strategy

To identify studies pertaining to the clinical results about HA versus THA in the treatment of FNF, we reviewed the Cochrane, Pubmed, Embase databases for relevant articles published through August 2019. We also reviewed the references of all identified articles to identify additional studies. Search terms were as follows: femoral neck fracture, femoral fracture, fracture of the femoral neck, FNF, hemihip arthroplasty, hemiarthroplasty, HA, THA, total hip arthroplasty, total hip replacement, total hip joint replacement. These terms were used in combination with “AND” or “OR”. This literature review was performed independently by two investigators, with a third resolving any disputes as needed.

Following the PICOS (Participants, Interventions, Comparisons, Outcomes and Study design) principle, the key search terms included (P) patients with FNF; (I) patients were treated by HA or THA; (C/O) the outcomes including complication, blood loss, surgery time, length of hospital, mortality, dislocation, reoperation, infection, pneumonia, pulmonary embolism, myocardial infarct, renal failure; (S) RTC, cohort study, or case-control study.

### Study selection criteria

Included studies met the following criteria: (1) randomized controlled trials, cohort studies or case-control studies; (2) the research objects are patients with FNF; (3) the treatment of experiment group is HA, the inventions of control groups are THA; (4) English or Chinese language.

Studies were excluded for meeting the following criteria: (1) repeat articles or results; (2) clear data errors; (3) case reports, case-control studies, theoretical research, conference reports, systematic reviews, meta-analyses, and other forms of research or comment not designed in a randomized controlled manner; (4) irrelevant outcomes; (5) has no control group.

Two investigators independently determined whether studies met with inclusion criteria, with a third resolving any disputes as needed.

### Data extraction and quality assessment

For each included study, two categories of information were extracted: basic information and primary study outcomes. Basic information relevant to this meta-analysis included author names, year of publication, sample size, age, gender, and study design. Primary clinical outcomes relevant to this analysis included: complication, blood loss, surgery time, length of hospital, mortality, dislocation, reoperation, infection, pneumonia, pulmonary embolism, myocardial infarct, renal failure. This data extraction was performed independently by two investigators, with a third resolving any disputes as needed.

### Statistical analysis

STATA v10.0 (TX, USA) was used for all analyses. Heterogeneity in study results was assessed using chi-squared and *I*^2^ tests and appropriate analysis models (fixed-effect or random-effect) were determined. A chi-squared *P* ≤ 0.05 and an *I*^2^ > 50% indicated high heterogeneity and a random-effects model was used in this case. A chi-squared *P* > 0.05 and an *I*^2^ ≤ 50% indicated acceptable heterogeneity and a fixed-effects model were instead used. Continuous variables are given as means ± standard deviations and were compared on the basis of mean difference (MD), while categorical data is given as percentages and compared based on relative risk (RR)/odds ratios (ORs). MD and 95% CI were used to analyze blood loss, surgery time, and length of hospital. The other indexes were analyzed by RR and 95% CI.

## Results

### Overview of included studies

**Fig. 1:**
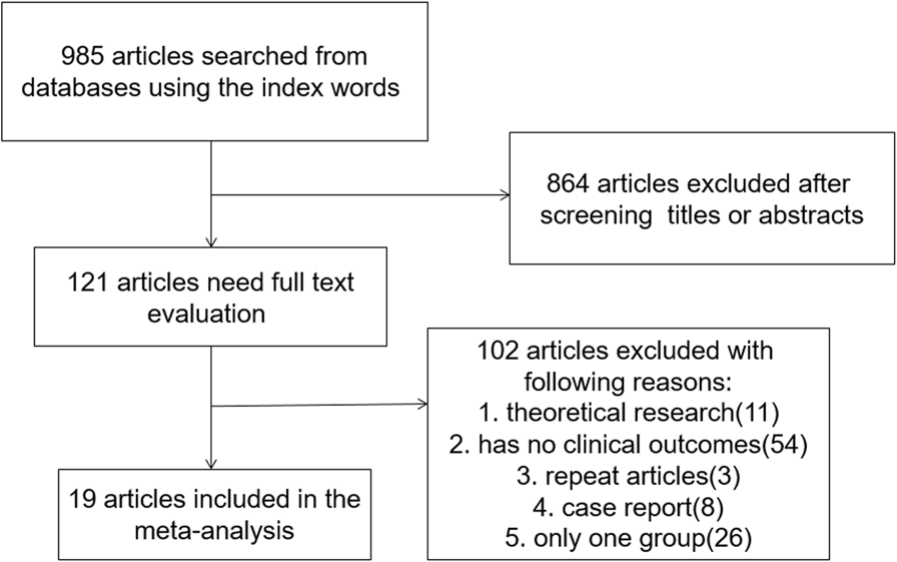
Literature search and selection strategy

We reviewed a total of 985 articles identified by our initial keyword search, of which 864 were excluded following title/abstract review. The PRISMA 2009 flow diagram has presented the detailed literature screening process. The PRISMA 2009 checklist has presented the specific framework and content of the article. The remaining 121 articles were subject to a complete full-text assessment, leading to 102 articles being excluded for failing to meet study inclusion criteria. Reasons for exclusion of these studies included theoretical research (11), no clinical outcomes (54), repeated articles (3), case reports (8), and only one group (26). We ultimately identified a total of 19 studies [6–24] that met with inclusion criteria for this meta-analysis, incorporating 413,140 patients in the HA group and 44,973 in the THA group. Study selection is outlined in Fig. 1.

Table 2 summarizes the basic information for each study, including author names, year of publication, sample, age, gender, and study design. The main age of all included studies was over 58 years old.

**Table 2:** The basic characteristics description of included studies

Study	No. of patients		Age		Gender		Study design
	HA	THA	HA	THA	HA	THA	
Sebastian Mukka 2019 [6]	118	61					Prospective, single-center cohort study
Ghazi Chammout 2019 [7]	60	60	86	85	45F	45F	RCT
Skender Ukaj 2019 [8]	47	47	77.64	78.11	15F	24F	Randomized, prospective, comparative interventional single-blinded study
Bheeshma Ravi 2019 [9]	2689	2689	79	79	1901F	1901F	A population-based, retrospective cohort study
Bheeshma Ravi 2019 [9]	26408	2713	83	79	19084F	1916F	A population-based, retrospective cohort study
Scott M Eskildsen 2018 [10]	275439	26017					A national for-fee database of Medicare patient procedure and diagnosis records from 2005 to 2012
Joey P. Johnson 2019 [11]	33660	9909	58.5	58.2	20533F	6480F	Cohort study
Fatih Cansah Barıshan 2018 [12]	22	16	76.9	73.6	14F	11F	Case-control
B. Boukebous 2017 [13]	101	98	83.3	77.8	28M	28M	Retrospective Case-control study
M. FUCHS 2017 [14]	70	91	81.7	76.4			Cohort study
Yong Tae Kim 2018 [15]	84	84	72.9	73.1	27M	26M	Retrospective cohort study
Stefan Bartels 2017 [16]	1030	572	64.9	63.7	672F	412F	Cohort study
Zhong Wang 2017 [17]	69142	1100	18115 M	304 M			Cohort study
Emmanouil Liodakis 2016 [18]	3192	866	81.9	72.9	952M	275M	Case-control
Christopher P. Miller 2014 [19]	783	419	544F	250F			Cohort study
Kyung-Soon Park 2013 [20]	41	44	71.6	73.6	14M	11M	Case-control
YUE-JU LIU 2012 [21]	55	60					Case-control
Carl Johan Hedbeck 2011 [22]	60	60	80.7	80.5	54F	47F	RCT
William Macaulay 2008 [23]	17	23	82	77	7F	14F	Case-control
James S. Gebhard 1999 [24]	122	44	76.2	75.2			Case-control

### Complication

**Fig. 2:**
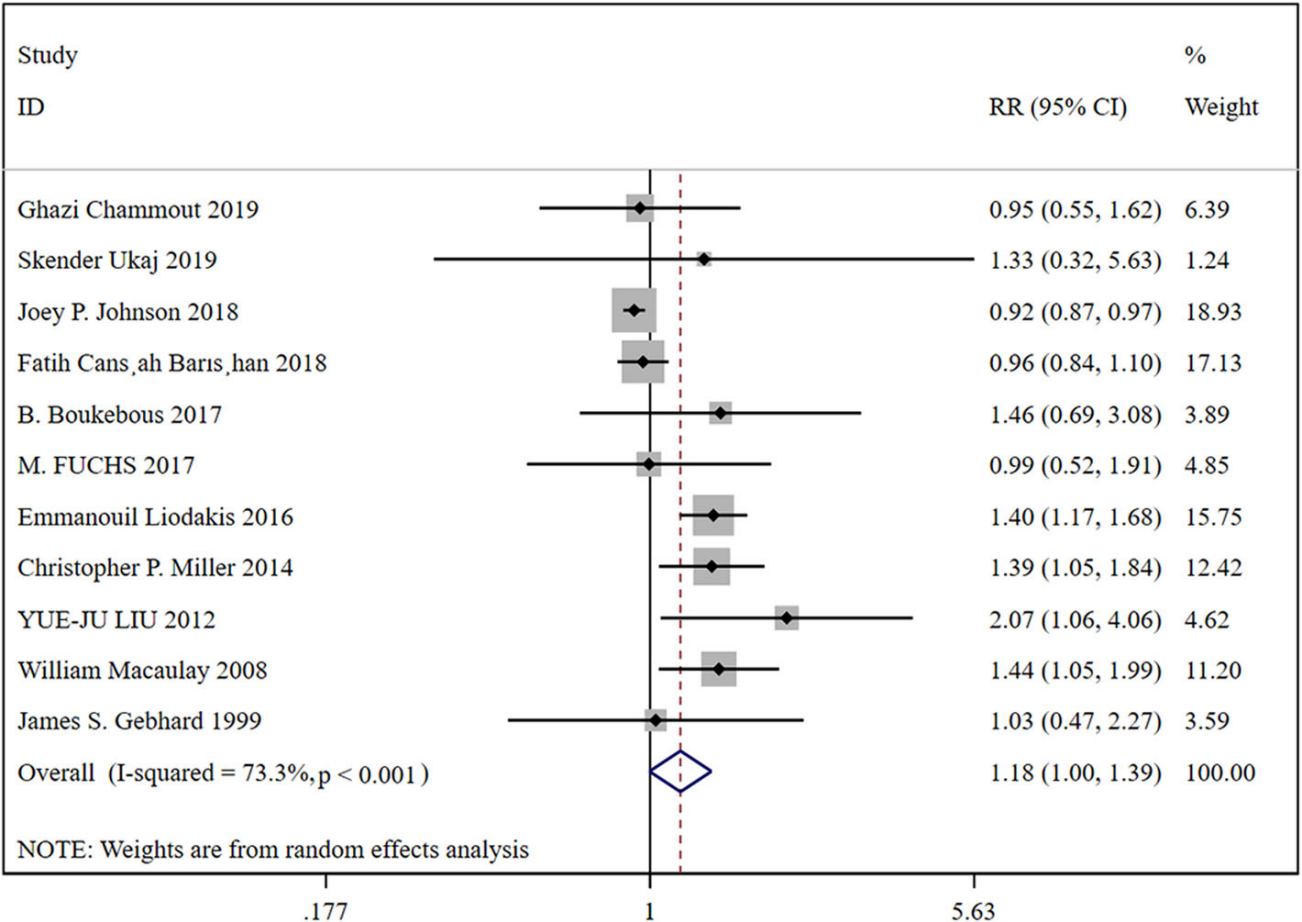
Forest plot for the complication

In total, 11 studies were included, containing 38,129 patients in the HA group and 11,633 patients in the THA group. Based on a chi-squared *P* = 0.000 < 0.05 and an *I*^2^ = 73.3% > 50%, a random-effects model was chosen to assess complication. The incidence of complication has no significant difference between the two groups (RR, 1.18; 95% CI, 1.00 ~ 1.39). The results were presented in Fig. 2.

### Blood loss

**Fig. 3:**
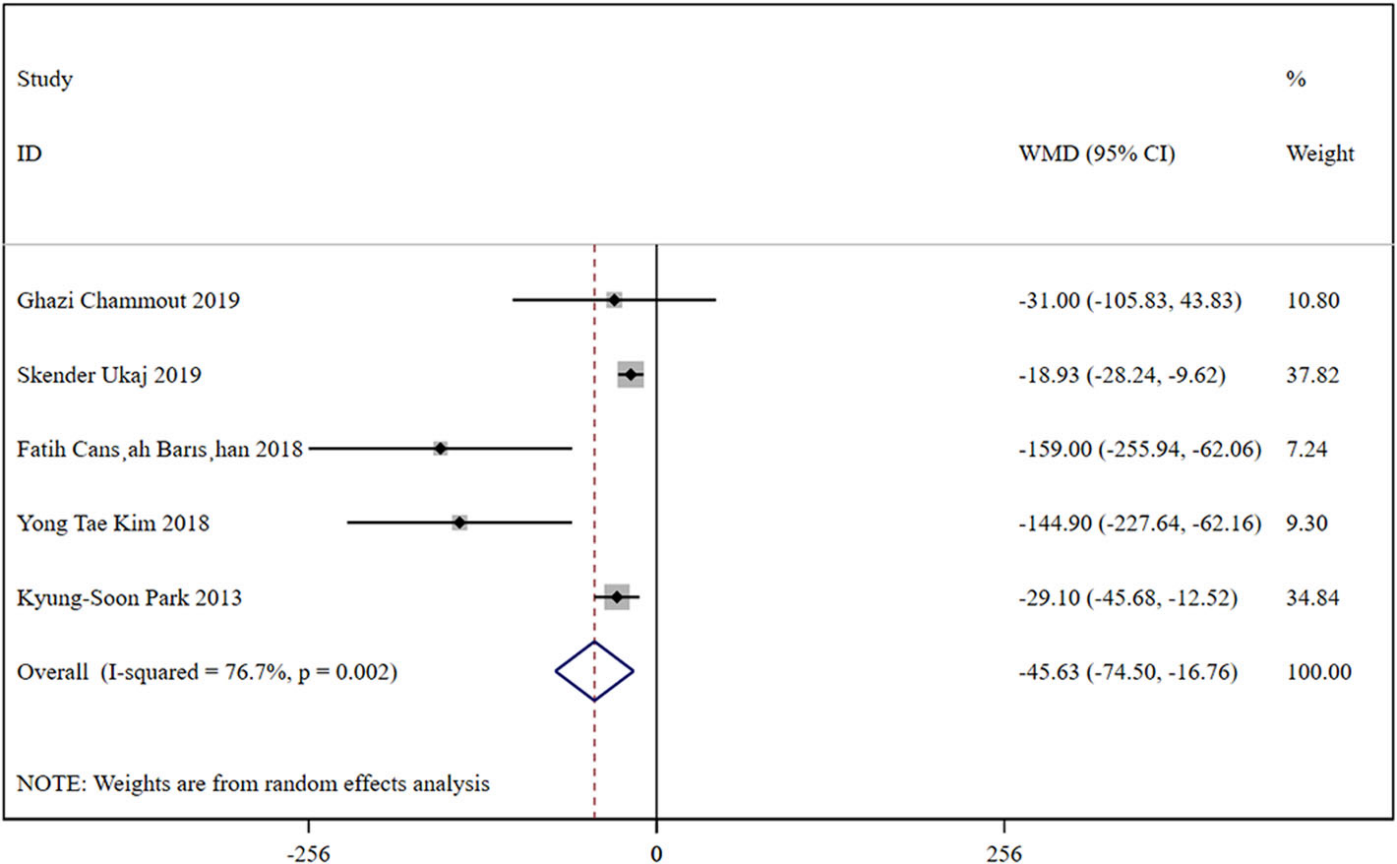
Forest plot for the blood loss

In total, 5 studies were included, containing 254 patients in the HA group and 251 patients in the THA group. Based on a chi-squared *P* = 0.002 < 0.05 and an *I*^2^ = 76.7% > 50%, a random-effects model was chosen to assess blood loss. The blood loss was significant lower in the HA group than the THA group (WMD, −45.63; 95% CI, −74.50 ~ −16.76). The results were presented in Fig. 3.

### Surgery time

**Fig. 4:**
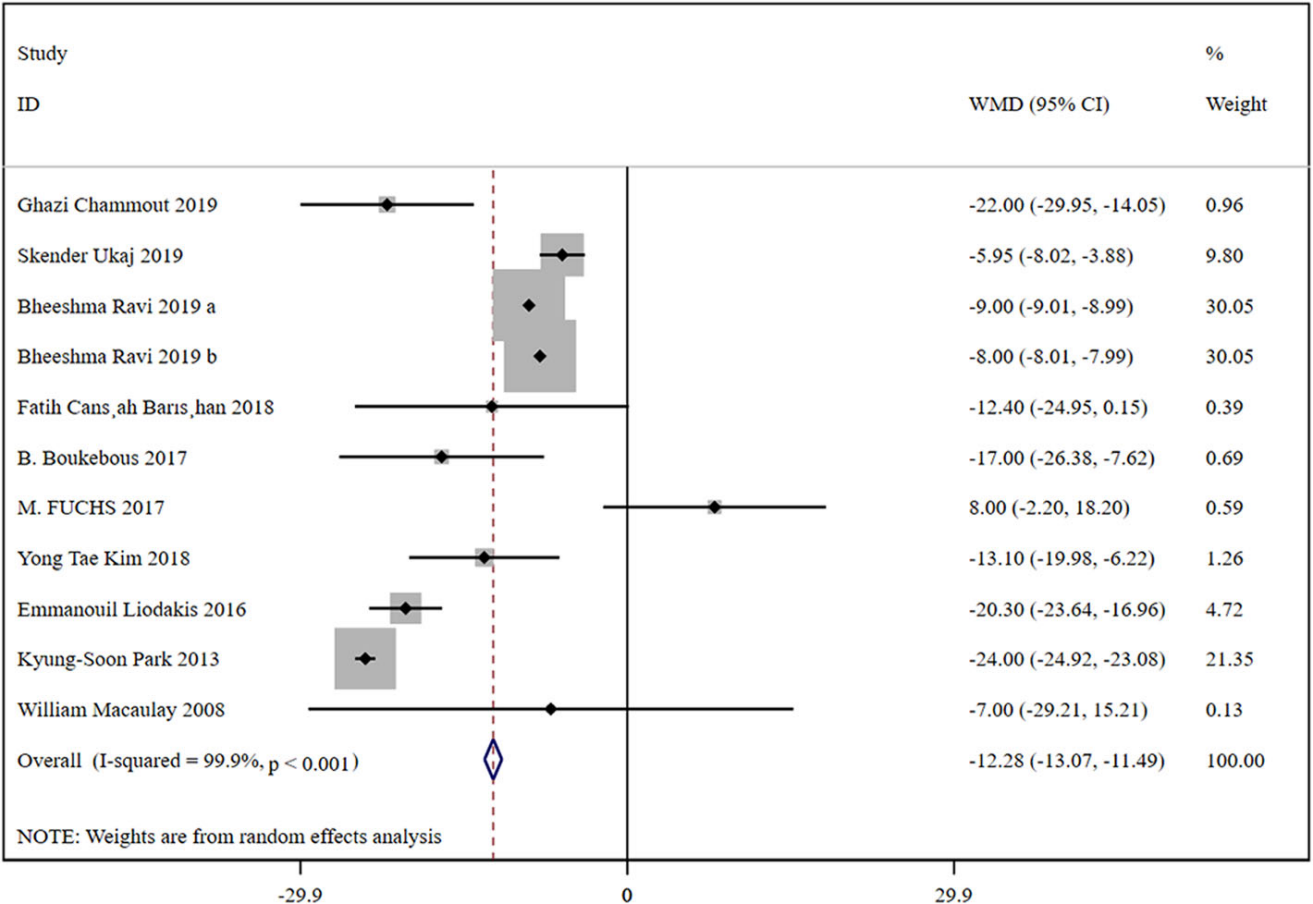
Forest plot for the surgery time

In total, 10 studies were included, containing 32,731 patients in the HA group and 6731 patients in the THA group. Based on a chi-squared *P* = 0.000 < 0.05 and an *I*^2^ = 99.9% > 50%, a random-effects model was chosen to assess surgery time. The surgery time was significantly decreased in the HA group than THA group (WMD, −12.28; 95% CI, −13.07 ~ −11.49). The results were presented in Fig. 4.

### Length of hospital

**Fig. 5:**
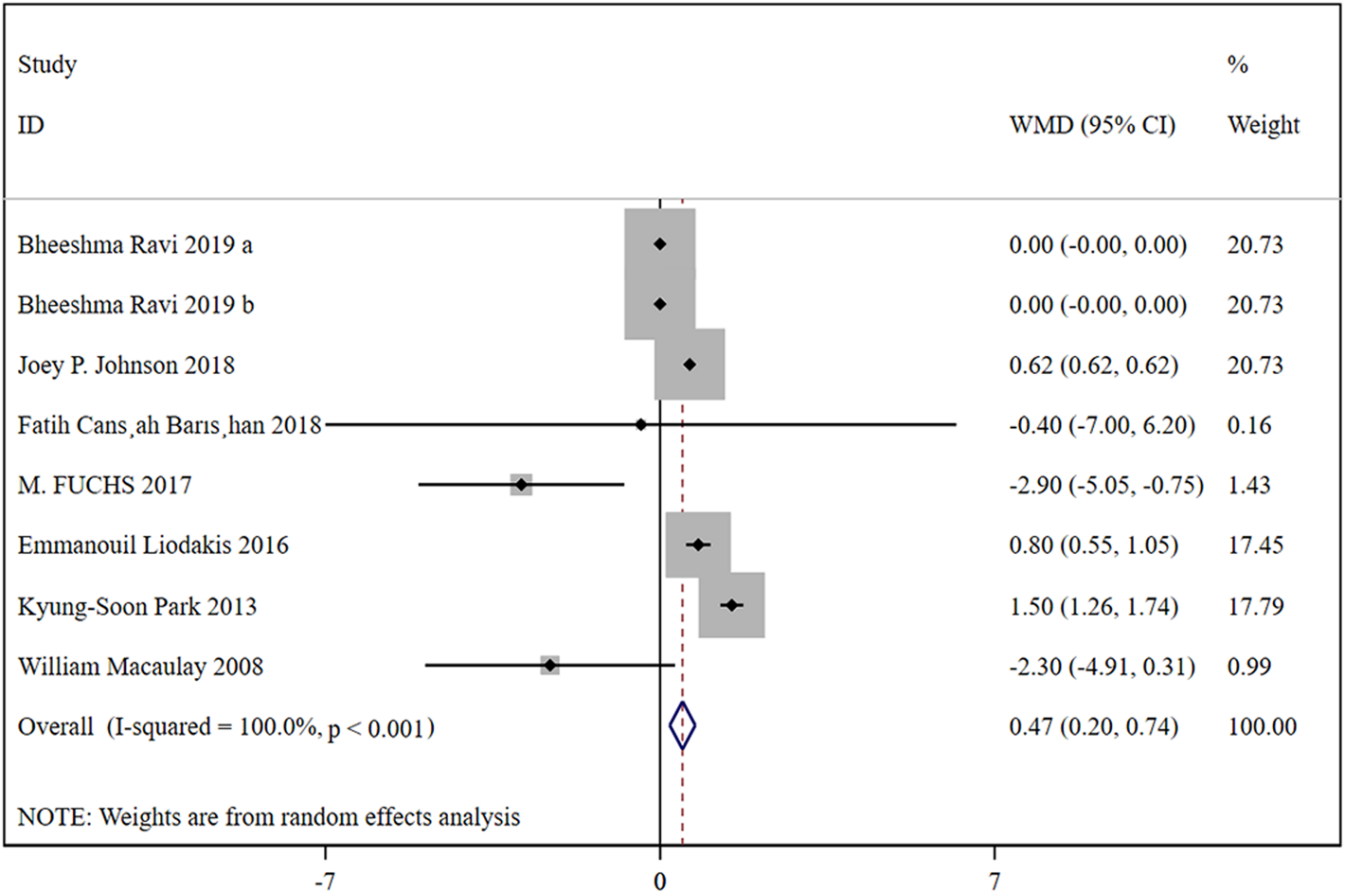
Forest plot for the length of hospital

In total, 7 studies were included, containing 66,099 patients in the HA group and 16,351 patients in the THA group. Based on a chi-squared *P* = 0.000 < 0.05 and an *I*^2^ = 100.0% > 50%, a random-effects model was chosen to assess the length of hospital. The length of hospital was significantly longer in the HA group than the THA group (WMD, 0.47; 95% CI, 0.20 ~ 0.74). The results were presented in Fig. 5.

### Mortality

**Fig. 6:**
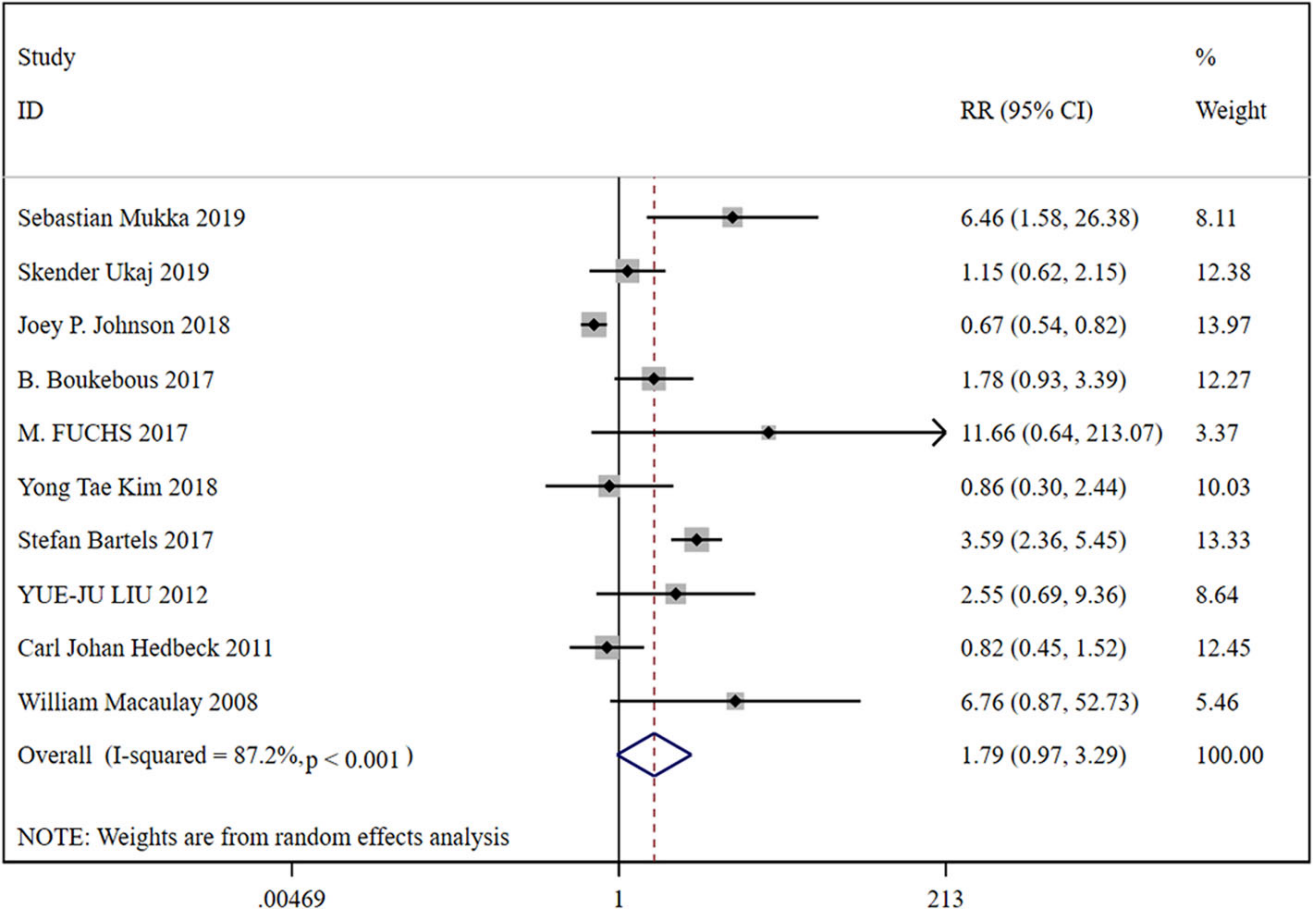
Forest plot for the mortality

In total, 10 studies were included, containing 35,242 patients in the HA group and 11,005 patients in the THA group. Based on a chi-squared *P* = 0.000 < 0.05 and an *I*^2^ = 87.2% > 50%, a random-effects model was chosen to assess mortality. The mortality has no significant difference between the two groups (RR, 1.79; 95% CI, 0.79 ~ 3.29). The results were presented in Fig. 6.

### The other indexes

The incidence of reoperation (RR, 1.319; 95% CI, 0.710 ~ 2.448), infection (RR, 1.037; 95% CI, 0.760 ~ 1.416), pulmonary embolism (RR, 1.160, 95% CI, 0.803 ~ 1.676), myocardial infarct (RR, 1.459; 95% CI, 0.988 ~ 2.156) has no significant difference between the two groups. The incidence of dislocation was significant decrease in the HA group than the THA group (RR, 0.536; 95% CI, 0.502 ~ 0.573). The incidence of pneumonia (RR, 1.511; 95% CI, 1.275 ~ 1.791) and renal failure (RR, 1.493; 95% CI, 1.188 ~ 1.876) were significantly higher in the HA group than the THA group. All the above results were presented in Table 3.

**Table 3:** The results of meta-analysis

Index	*N* (case/control)	*RR* (95% CI)	P^*^	*I*^*2*^	*P*^*#*^	*P* value	
						Begg’s	Egger’s
Dislocation	374242/33026	0.536 (0.502, 0.573)	0.098	35.5%	0.000	0.951	0.032
Reoperation	378085/34168	1.319 (0.710, 2.448)	0.000	91.9%	0.381	0.602	0.257
Infection	341683/42513	1.037 (0.760, 1.416)	0.000	82.4%	0.819	0.283	0.113
Pneumonia	32621/6546	1.511 (1.275, 1.791)	0.415	2.3%	0.000	0.754	0.457
Pulmonary embolism	33241/6877	1.160 (0.803, 1.676)	0.394	4.6%	0.429	0.386	0.154
Myocardial infarct	32458/6458	1.459 (0.988, 2.156)	0.740	0.0%	0.058	0.368	0.150
Renal/kidney failure	32441/6435	1.493 (1.188, 1.876)	0.730	0.0%	0.001	0.133	0.173

^*^P value of heterogeneity chi-squared

^#^*P* value of pooled statistic

### Quality and bias assessment

**Fig. 7:**
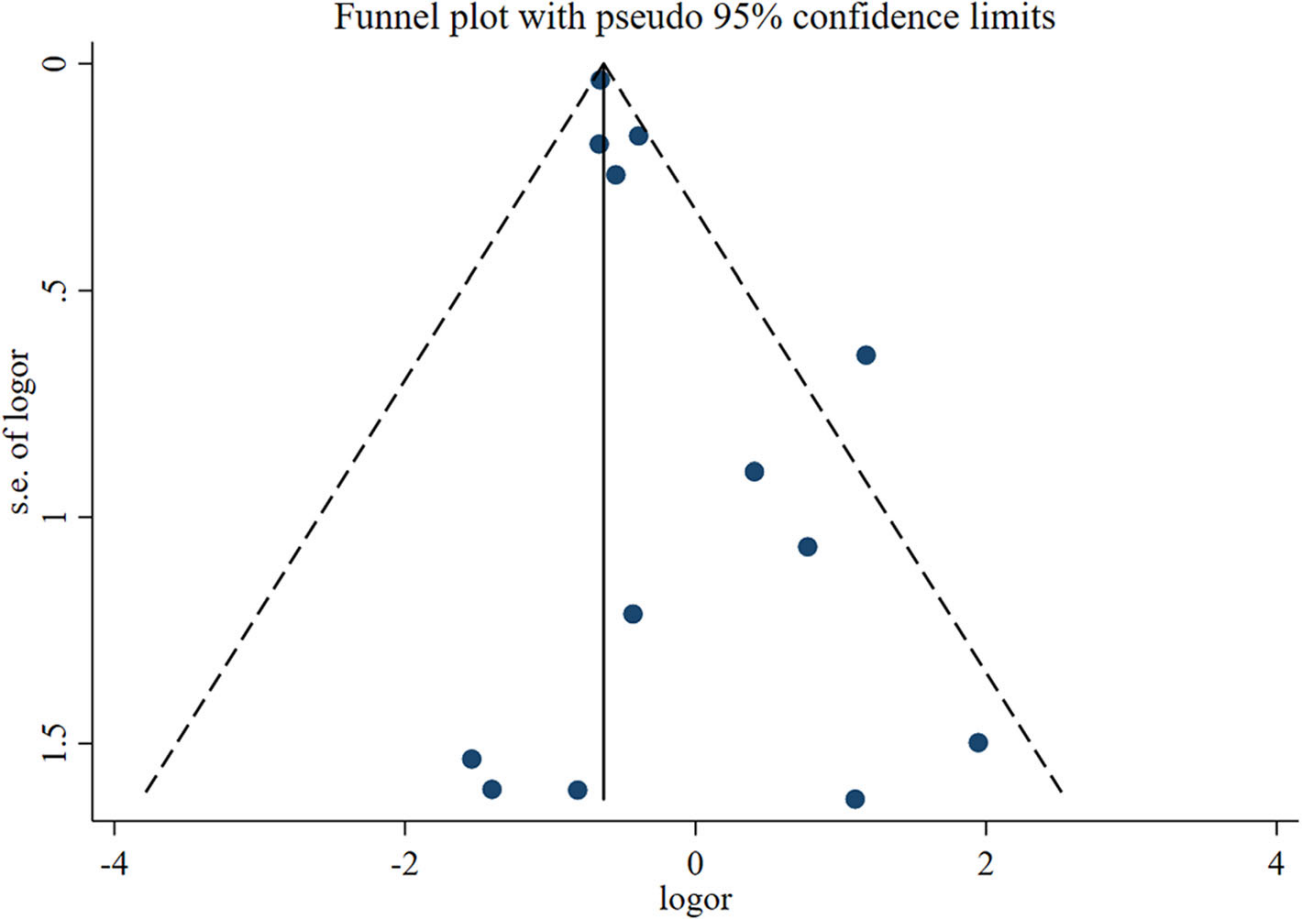
Funnel plot analysis of included studies

An assessment of study quality and risk of bias was performed using multiple complementary methods including funnel plots, Begg’s and Mazumdar’s rank test, and Egger’s test. There was clear symmetry in the log RR funnel plot for dislocation for these studies, suggesting a low publication bias risk (Fig. 7). The results of Begg’s and Mazumdar’s rank test (*Z* = 1.25, *p* = 0.210) and Egger’s test (*P* = 0.072) both suggested that there was not any significant risk of bias among study results.

## Discussion

FNF mostly occurs in middle-aged and elderly people and is associated with decreased bone quality due to osteoporosis. Fractures can occur when a mild twisting force is experienced, most often as a result of a fall while walking or slipping. When falling, as a result of the body torsion, indirect transmission of violence resulting in FNF. FNF are less common in adolescents, and when they do occur, they often require significant violence and are often unstable. The two main treatments for FNF are internal fixation and joint replacement. The physical characteristics of the elderly are relatively low-stress response ability to trauma, relatively weak immune suppression, and defense function. Fracture healing in the elderly is relatively slow, and most of the elderly combined with cardiopulmonary and brain diseases. Therefore, elderly patients who are bedridden for a long time are more likely to suffer from serious complications, such as some diseases such as urinary tract infection, lung inflammation, and bedsore. Elderly patients are in bed for a long time, and due to the factors of fracture, the disease existing before the fracture is more prone to relapse, leading to a high disability rate and mortality rate.

Currently, there are many surgical methods to treat FNF, but generally speaking, it is generally agreed that the better method is artificial joint replacement. Currently, HA and THA are widely used, and both advantages and disadvantages exist in clinical practice. Hip replacement can significantly reduce the incidence of postoperative joint pain in patients, and because of the patient’s early weight-bearing walking, it is very important to maintain the muscle strength of the affected limb and restore good overall health. The advantages and disadvantages were discussed in the following aspects.

Firstly, it is about the risk of operation. Compared with HA, THA has many disadvantages, such as complex, large trauma, long operation time, and more intraoperative and postoperative bleeding. The advantages of HA include less trauma, short operation duration, small blood loss, simple operation compared with total hip, and high surgical safety compared with THA. However, with the development of all aspects of science and technology, the surgical risk of THA is becoming less and less, because the differences between the two surgical methods in many aspects become smaller. However, the surgical risk of THA is still high in the treatment of elderly patients with medical complications. In our study, we found that the blood loss (WMD, −45.63; 95% CI, −74.50 ~ −16.76), surgery time (WMD, −12.28; 95% CI, −13.07 ~ −11.49) were all significant decreased in the HA group than the THA group. However, the length of hospital was significantly longer in the HA group than the THA group (WMD, 0.47; 95% CI, 0.20 ~ 0.74). The mortality has no significant difference between the two groups (RR, 1.79; 95% CI, 0.79 ~ 3.29).

Secondly, it is about the reoperation. The rate of reoperation is also an important factor affecting the choice of hip arthroplasty. Dislocation of prosthesis, wear of acetabular cartilage, and sterility of artificial prosthesis are the main causes of reoperation. After a hip replacement, the joint is removed and replaced with a new one. The procedure is relatively complex and technically demanding. This operation is definitely not a simple “old for new” process. It also takes into account the differences in surgical approaches used by surgeons during the first operation, the changes in anatomy and surrounding soft tissue, and the bone defects caused by the first artificial prosthesis. Therefore, for patients, it is necessary to give them good satisfaction and reduce the probability of reoperation. In our study, we found that the incidence of reoperation (RR, 1.319; 95% CI, 0.710 ~ 2.448) has no significant difference between the two groups. The incidence of dislocation was significant decrease in the HA group than THA group (RR, 0.536; 95% CI, 0.502 ~ 0.573).

Third, it is about the complications. The elderly, as a group with a high incidence of FNF, account for about 46 percent of fractures in the elderly (4%). Elderly people suffer from many basic diseases, weakened constitution, decreased organ function, and surgical trauma, so the postoperative recovery is slow and the postoperative bed rest time is long. If the risk of postoperative complications increases, it will have a certain impact on the efficacy and prognosis of the operation. The incidence of complication has no significant difference between the two groups (RR, 1.18; 95% CI, 1.00 ~ 1.39). The infection (RR, 1.037; 95% CI: 0.760 ~ 1.416), pulmonary embolism (RR, 1.160; 95% CI, 0.803 ~ 1.676), myocardial infarct (RR, 1.459; 95% CI, 0.988 ~ 2.156) has no significant difference between the two groups. The incidence of pneumonia (RR, 1.511; 95% CI, 1.275 ~ 1.791) and renal failure (RR, 1.493; 95% CI, 1.188 ~ 1.876) were significantly higher in the HA group than the THA group.

However, there are certain limitations to the present analysis, which are as follows: (1) the limited number of included studies; (2) individual studies had variations in exclusion/inclusion criteria; (3) surgical skills varied between studies; (4) the severity of FNF in patients varied between studies; (5) the background diseases of patients were various between studies; (6) some studies were not high-quality; (7) pooled data were analyzed, as individual patient data was not available, precluding more in-depth analyses.

## Conclusion

In a word, we found that HA has favor in decrease blood loss and surgery time and THA has favor in decrease the length of hospital, the incidence of pneumonia and renal failure. For the selection of surgical methods for femoral neck fracture in the elderly, we should consider several aspects, such as the age of the patient, whether there is osteoporosis, the type of femoral neck fracture, the preoperative reduction situation, and the needs of the patient and his family for the postoperative situation.

## Supplementary Information

**Additional file 1.**

**Additional file 2.**

## Data Availability

The datasets used and/or analyzed during the current study are available from the corresponding author on reasonable request.

## Abbreviations

HA: Hemiarthroplasty
THA: Total hip arthroplasty
FNF: Femoral neck fractures
ORs: Odds ratios
MD: Mean difference
95% CI: 95% confidence interval
