# Supplementary material for: Hemiarthroplasty compared to total hip arthroplasty for the treatment of femoral neck fractures: a systematic review and meta-analysis
Source: J Orthop Surg Res. 2021 Mar 3;16:172. doi: 10.1186/s13018-020-02186-4 (PMC7931515; doi:10.1186/s13018-020-02186-4)
Supplement: Supplementary file 1 — Additional file 1. [file 13018_2020_2186_MOESM1_ESM.doc]

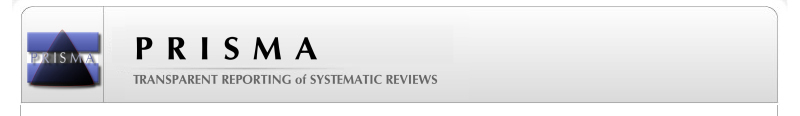
**PRISMA 2009 Flow Diagram**

**Screening**

**Included**

**Eligibility**

**Identification**

Records identified through database searching
(n =976)

Additional records identified through other sources
(n = 9)

Records after duplicates removed
(n = 512 )

Records screened
(n = 389 )

Records excluded
(n = 123 )

Full-text articles assessed for eligibility
(n = 121)

Full-text articles excluded, with reasons
(n = 96 )

Studies included in qualitative synthesis
(n = 25 )

Studies included in quantitative synthesis (meta-analysis)
(n = 19 )
